# Supplementary material for: Cryo-EM reveals structural breaks in a patient-derived amyloid fibril from systemic AL amyloidosis
Source: Nat Commun. 2021 Feb 8;12:875. doi: 10.1038/s41467-021-21126-2 (PMC7870857; doi:10.1038/s41467-021-21126-2)
Supplement: Supplementary file 1 — Supplementary Information [file 41467_2021_21126_MOESM1_ESM.pdf]

Supplementary Information

Cryo-EM reveals structural breaks in a patient-derived amyloid fibril from systemic AL amyloidosis

L. Radamaker et al.

Supplementary Figure 1

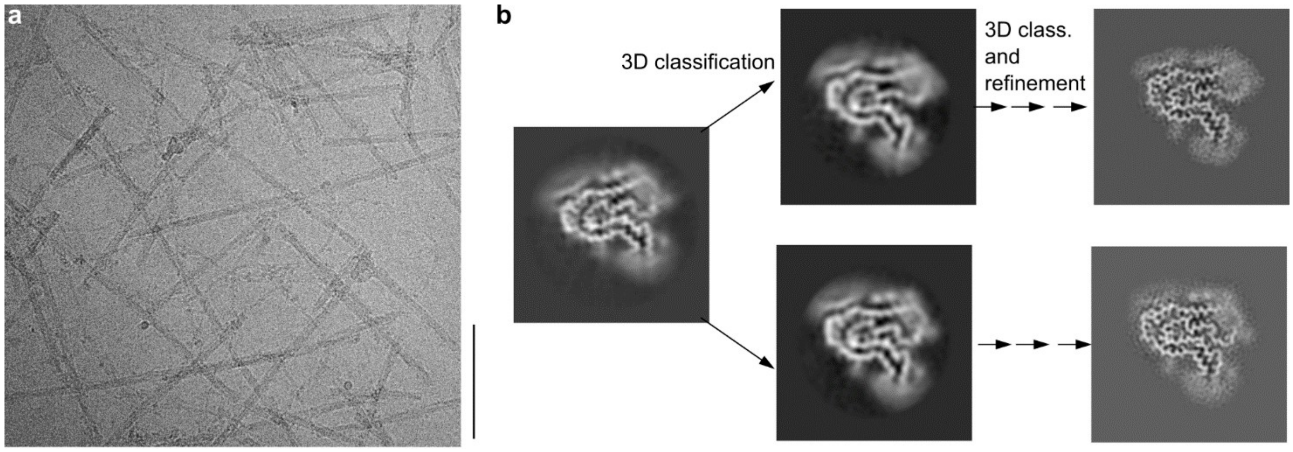

Supplementary Fig. 1

Work flow of steps of the reconstruction process.

(a) Representative cryo-EM image of the extracted fibrils. Scale bar: 100 nm. In total, 1,390 micrographs were analyzed from one fibril sample. (b) Schematic illustration of the workflow. Based on an initial reconstruction, 3D classification separated two subsets exhibiting different density paths. Through several further classification and refinement steps, the final reconstructions were obtained.

Supplementary Figure 2

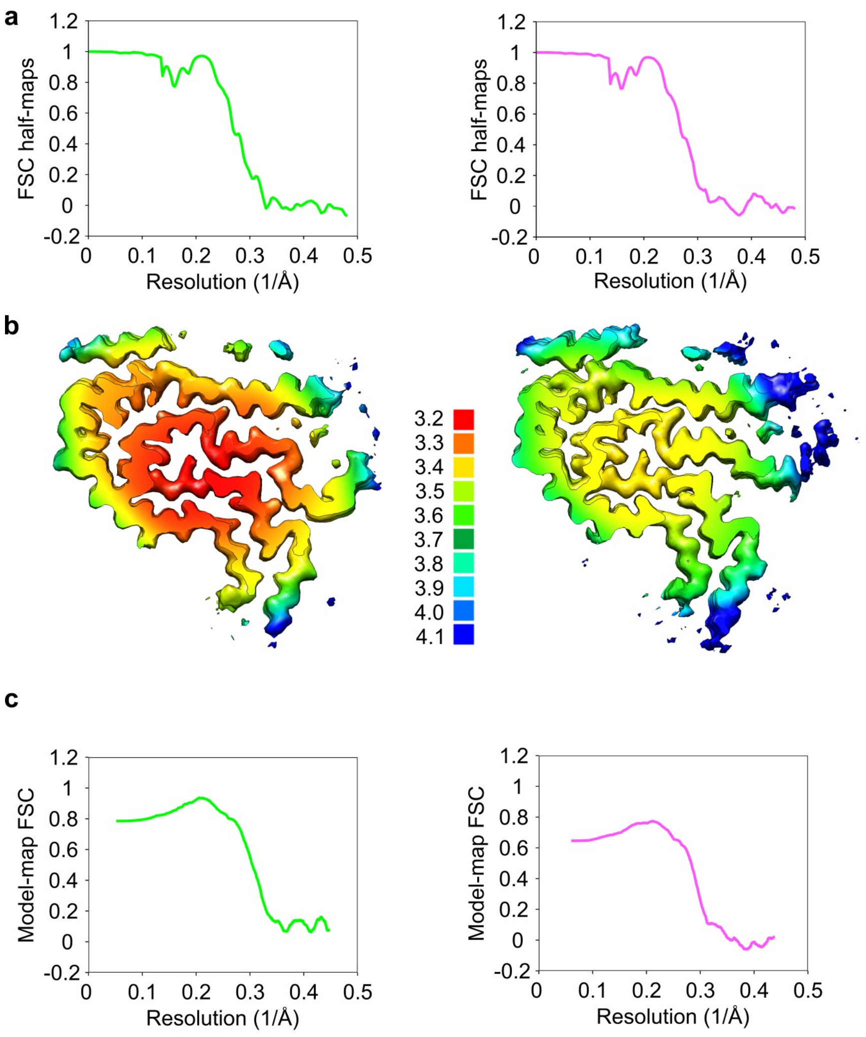

Supplementary Fig. 2

Map resolution and model fit.

(a) FSC curves for fibril structures A (green, left) and B (magenta, right). (b) Local resolution maps for A (left) and B (right). (c) Model-map FSC curves for models of conformation A (green, left) and B (magenta, right)

Supplementary Figure 3

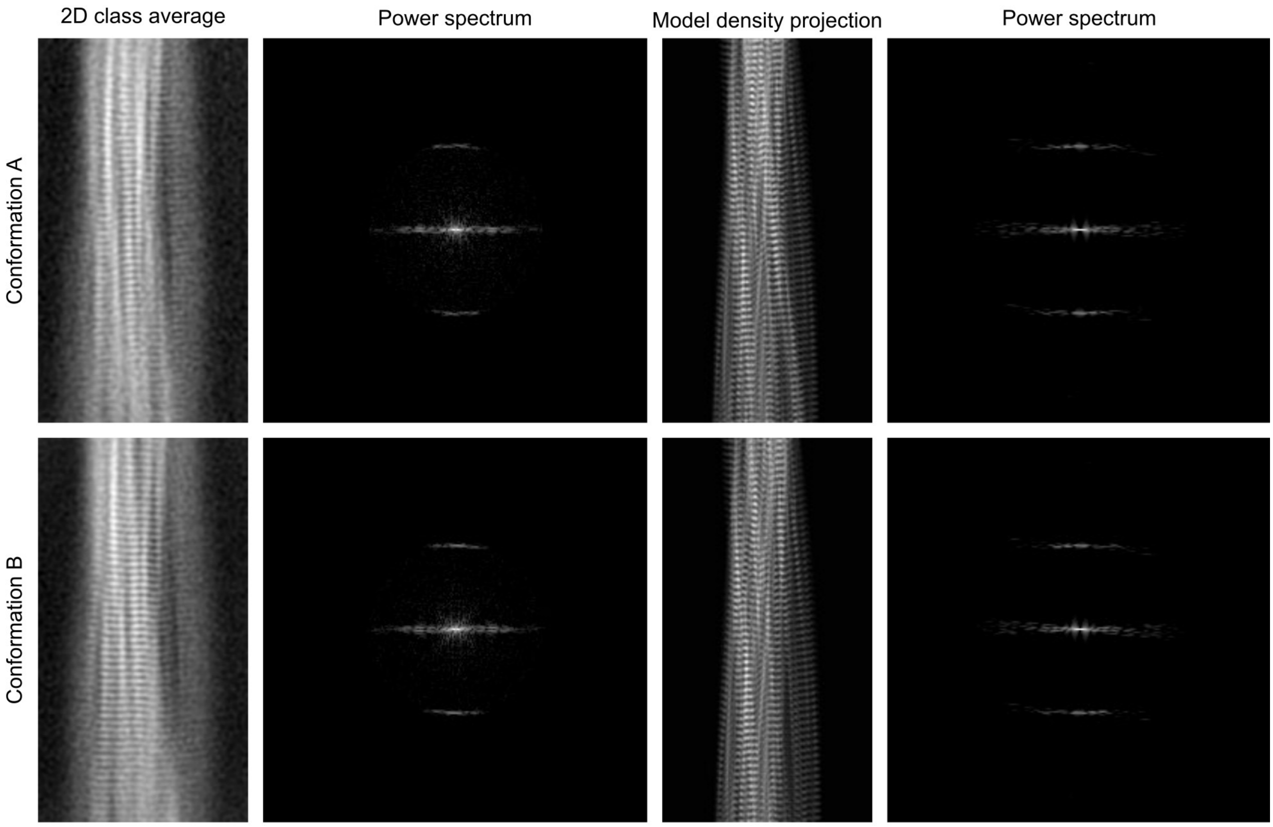

Supplementary Fig. 3

Comparison of the molecular models with 2D class averages.

For the two conformations A and B respectively, 2D class averages and model density projections corresponding to the same fibril regions are shown, with their respective power spectra. The length of the fibril section is approximately 266 Å.

Supplementary Figure 4

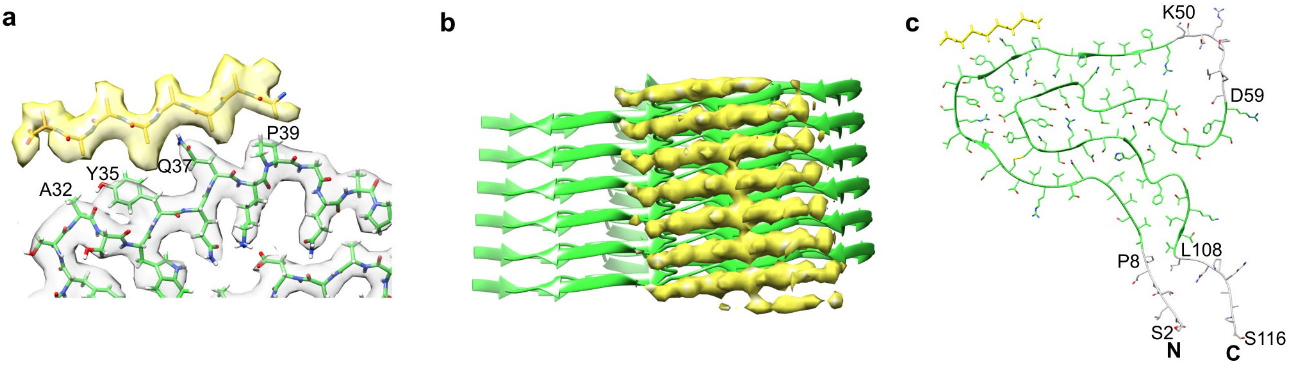

Supplementary Fig. 4

Defined extra density decorating the fibril surface.

(a) Enlarged view of the defined extra density (yellow) seen in structure A (green), overlayed with an 8-residue poly-L-alanine chain in parallel β-sheet conformation (yellow). (b) Side view of the defined extra density (yellow) next to the molecular model of a stack of fibril proteins in conformation A (ribbon diagram, green) showing the rise along the z-axis as well as the offset between the extra density and the fibril protein model. (c) Ribbon diagram including side chains of a single fibril protein layer in conformation A (green) showing all residues in the fibril protein including the disordered N- (Ser2-Pro8) and C-terminus (Leu108-Ser116) and the central disordered region (Lys50-Asp59) marked in gray, as well as the 8-residue poly-L-alanine chain as in (a) (yellow), representing the defined extra density. The gray segments are depicted in arbitrary conformation.

Supplementary Figure 5

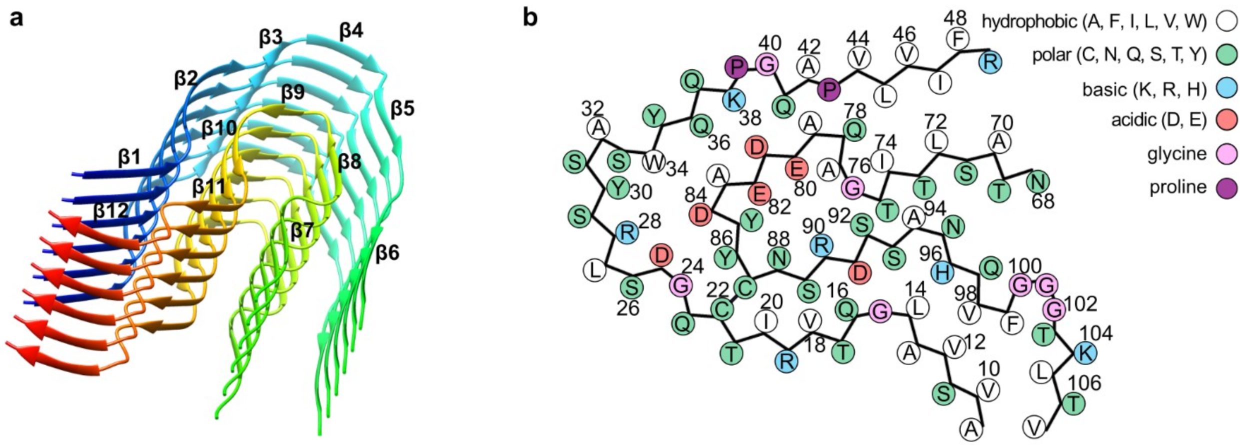

Supplementary Fig. 5

Structural features of fibril protein conformation B.

(a) Ribbon diagram of a stack of six fibril proteins in conformation B. β-strands have been colored in rainbow palette from the N- to C-terminus. (b) Schematic representation of the amino acid positions in conformation B. See Fig. 2b, c for the corresponding images showing conformation A.

Supplementary Figure 6

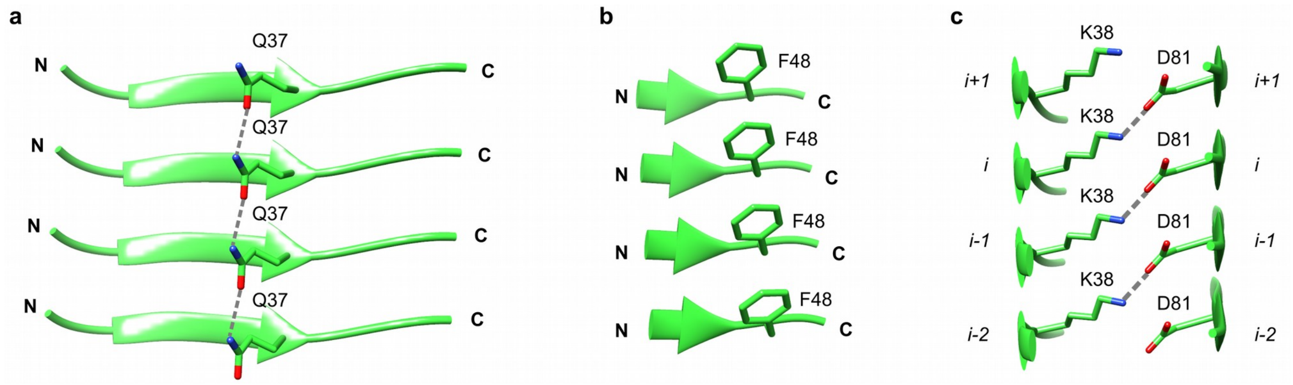

Supplementary Fig. 6

Side chain-side chain interactions across the layers of the fibril model.

(a) Stack of four fibril protein layers showing a polar ladder formed by Gln37 residues. (b) Four Phe48 residues in a configuration allowing for π-π (aromatic) stacking. (c) Electrostatic interactions between Lys38 of layer *i* and Asp81 of layer *i+1* demonstrating the interlocking of fibril protein layers. The images are based on a model of fibril structure A (green).

Supplementary Figure 7

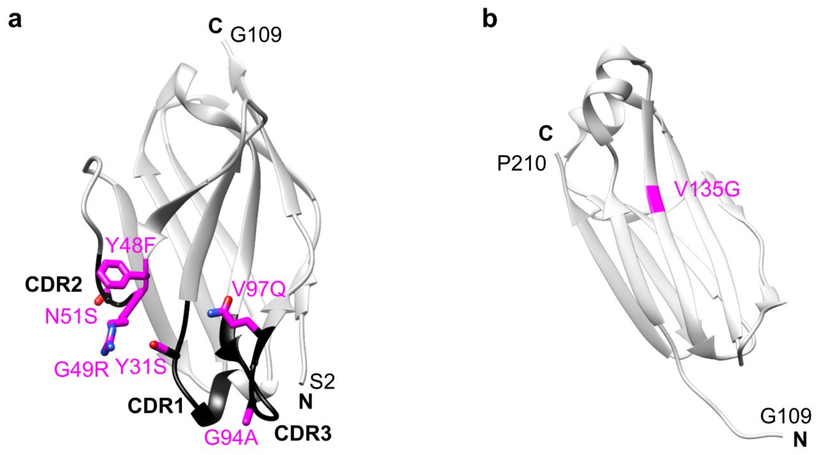

Supplementary Fig. 7

Location of the FOR005 mutations within the natively folded LC domains.

(a) Location of the mutations (magenta) and CDRs (black) within the structure of the refolded FOR005 fibril protein (PDB: 5L6Q[1]). (b) Location of the Val135Gly mutation (magenta), in the C_L domain of a homologous LC, containing an *IGLC2* GL segment (adapted from PDB: 4EOW[2] see methods for details). N- and C-terminal residues are marked.

Supplementary Figure 8

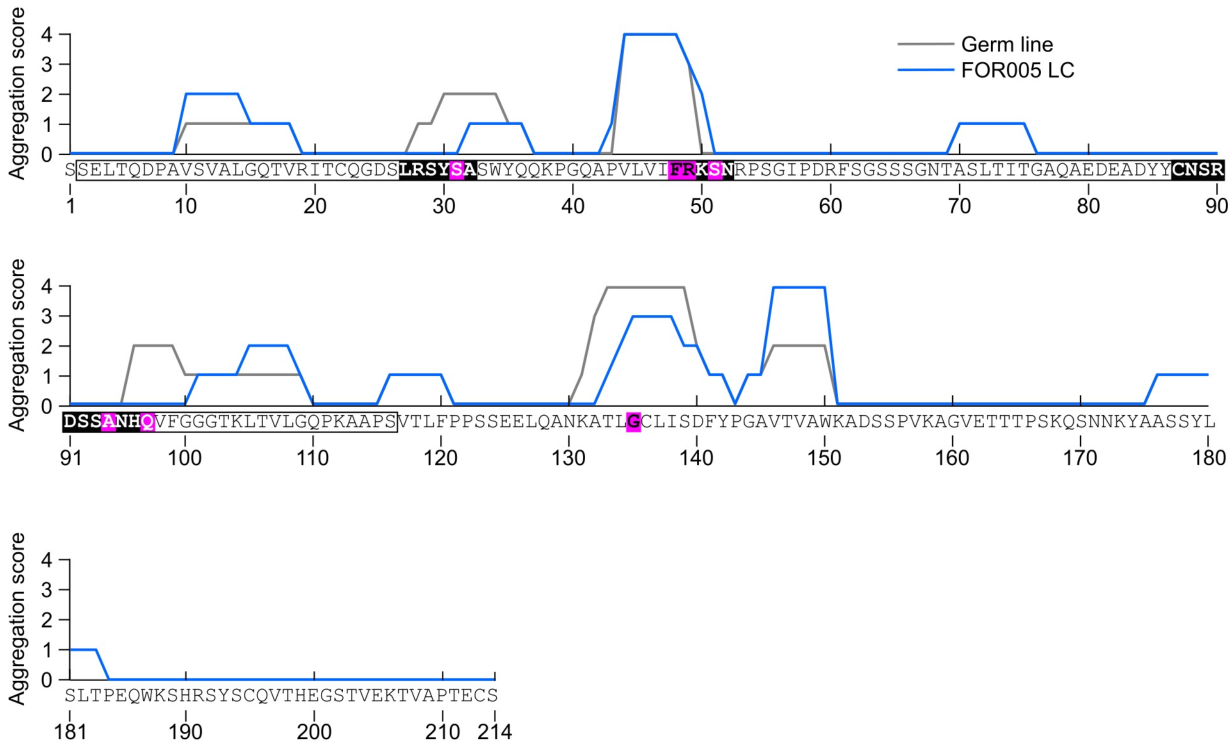

Supplementary Fig. 8

Aggregation score of the FOR005 precursor LC and of the corresponding GL protein sequence.

Aggregation score of the FOR005 LC precursor protein sequence (blue) and of the corresponding GL protein sequence (gray). The sequence on the x-axis is the FOR005 LC precursor protein sequence. The box denotes the range of the fibril protein (Ser2-Ser116). CDRs are indicated by white letters with black boxes. Mutations in the CDRs are indicated by white letters with magenta boxes. Mutations outside of the CDRs are indicated by black letters with magenta boxes.

Supplementary Figure 9

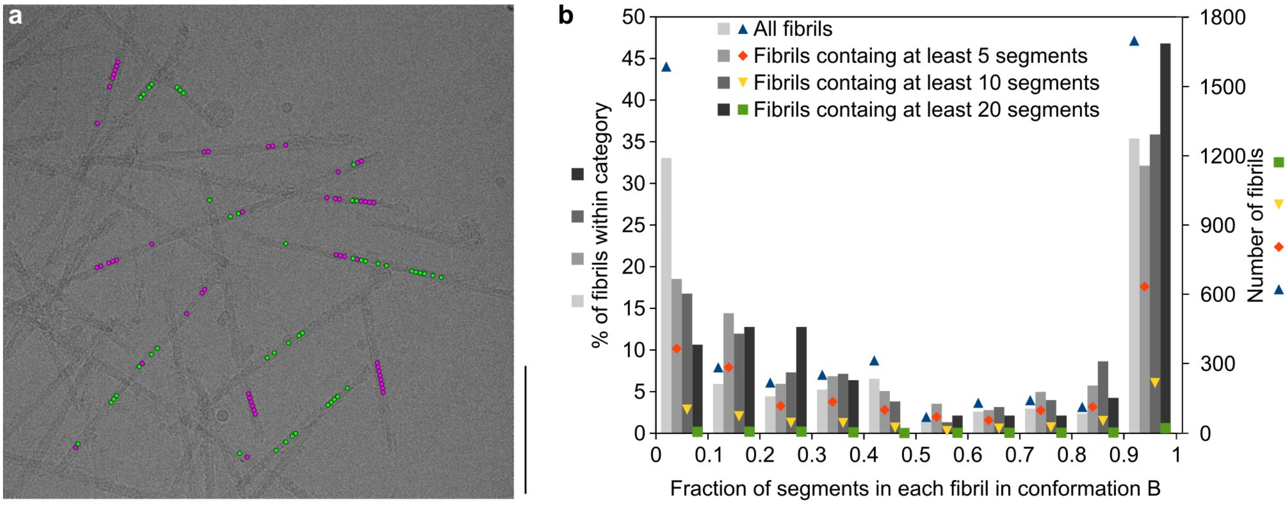

Supplementary Fig. 9

Evidence for structural breaks after the final round of 3D classification

(a) Representative cryo-EM micrograph showing the location of segments classified as fibril structure A (green) and fibril structure B (magenta) after the final round of 3D classification. Scale bar: 100 nm. Data were collected from 1,130 micrographs from one fibril sample. Figure 4a shows the same image, highlighting all the segments from the first round of 3D classification. (b) Histogram of the fraction of segments classified as fibril structure B, per fibril, after the final 3D classification. Different thresholds were chosen for the minimum number of segments per fibril, resulting in four categories: all fibrils (4,795 fibrils), fibrils containing a minimum of 5 segments (1,970 fibrils), fibrils containing a minimum of 10 segments (602 fibrils) and fibrils containing a minimum of 20 segments (47 fibrils). The percentages were normed using the total number of fibrils in each category. Colored points show the absolute number of fibrils in each category and group (fraction in conformation B). The data set (n = 23,124) contained 11,003 segments for fibril structure A and 12,122 segments for fibril structure B. Figure 4b shows an analogous histogram including all the segments from the first round of 3D classification.

Supplementary Figure 10

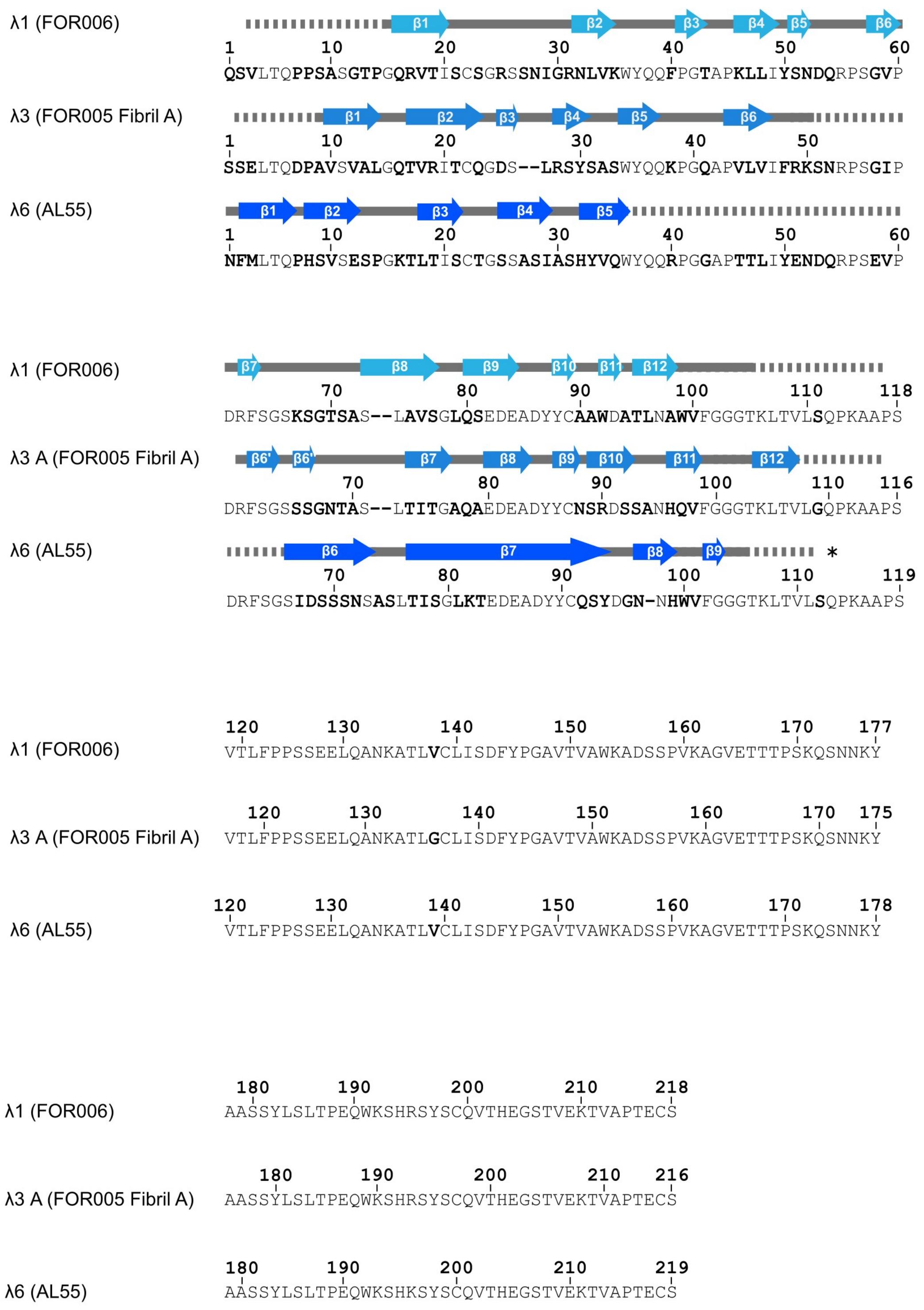

Supplementary Fig. 10

Sequence alignment and secondary structure comparison of ex vivo AL amyloid fibrils.

Sequence alignment of ex vivo AL amyloid fibril LCs for which a cryo-EM structures is available, combined with a schematic representation of the secondary structure of these fibrils. Non-conserved residues are marked in bold. Arrows indicate β-strands. Continuous lines indicate ordered conformation, dotted lines indicate disordered segments. Star: no single truncation site reported. The definition of secondary structural elements follows the definition in the respective manuscripts (FOR006[3]: light blue, FOR005: marine blue, AL55[4]: dark blue).

Supplementary Table 1

	Fibril A<br>(EMD-11031)<br>(PDB 6Z10)	Fibril B<br>(EMD-11030)<br>(PDB 6Z1I)
**Data collection and processing**		
Magnification	130,000	130,000
Voltage (kV)	300	300
Electron exposure (e– Å^{-2})	1	1
Defocus range (μm)	0.2 – 4.2	0.2 – 4.2
Pixel size (Å)	1.04	1.04
Symmetry imposed	C1	C1
Initial particle images (no.)	194,502	194,502
Final particle images (no.)	11,003	12,122
Map resolution (Å)<br>FSC threshold (0.143)	3.2	3.4
Rise (Å)	4.8	4.8
Twist	-1.1	-1.1
Pitch (nm)	155	155
**Refinement**		
Initial model used (PDB code)	De novo	PDB 6Z10
Model resolution (Å)		
FSC threshold 0.143	3.1	3.2
FSC threshold 0.5	3.4	3.5
Map CC (mask)	0.76	0.73
EMRinger ZScore	5.09	5.97
EMRinger Score	2.88	3.59
Model composition		
Non-hydrogen atoms	3,972	3,648
Protein residues	534	486
Ligands	0	0
B factors (Å^2)		
Protein	73.24	72.43
Ligand	-	-
R.m.s. deviations		
Bond lengths (Å)	0.009	0.009
Bond angles (°)	1.795	1.745
Validation		

MolProbity score	1.42	1.61
Clashscore	1.42	3.08
Poor rotamers (%)	0	0
Ramachandran plot		
Favored (%)	91.76	91.34
Allowed (%)	8.24	8.66
Disallowed (%)	0	0
B-factor after autosharpening (Å^2)	71.3	51.8
Contour level	6σ	6σ

Supplementary Table 1.

Reconstruction and modeling statistics.

Supplementary References

[1] Annamalai, K. et al. Common fibril structures imply systemically conserved protein misfolding pathways in vivo. *Angew Chem Int Ed* **56.26,** 7510-7514 (2017)

[2] Blech, M. et al. Molecular structure of human GM-CSF in complex with a disease-associated anti-human GM-CSF autoantibody and its potential biological implications. *Biochem J* **447.2,** 205-215 (2012)

[3] Radamaker, L., et al. Cryo-EM structure of a light chain-derived amyloid fibril from a patient with systemic AL amyloidosis. *Nat Commun* **10.1,** 1-8 (2019)

[4] Swuec, P., et al. Cryo-EM structure of cardiac amyloid fibrils from an immunoglobulin light chain AL amyloidosis patient. *Nat Commun* **10.1,** 1-9 (2019)
